# A modelling framework for improved design and decision-making in drug development

**DOI:** 10.1371/journal.pone.0220812

**Published:** 2019-08-28

**Authors:** Stig Johan Wiklund

**Affiliations:** Captario AB, Göteborg, Sweden; Shandong University of Science and Technology, CHINA

## Abstract

The development of a new drug is an extremely high-risk enterprise. The attrition rates of development projects and the average costs for each launched product are daunting, and the completion of a development program requires a very long time horizon. These facts imply that there are huge potential gains, should one be able to improve efficiency and enhance decision-making capabilities. In this paper, we argue that substantial gains can be achieved by adapting a holistic view of drug development. Historically, too much planning, design and decision-making in the pharmaceutical development has been based on locally optimising separate parts of the development program, and too often important sources of uncertainty are ignored. We propose instead a model-based approach built on two essential pillars; (1) an integrated holistic view of the development program, including post-launch marketing and sales, with all parts evaluated simultaneously; (2) an explicit appreciation of all relevant sources of uncertainty. Computer simulations are utilised to evaluate the properties of the program options at hand, and to provide valuable quantitative decision support. Applications of this modelling approach have proven to add large value to development projects in terms of better program options being generated and more value-adding decisions taken.

## Introduction

The decreasing level of productivity and the increased spending in research and development (R&D) has for a long time been an issue of concern in the pharmaceutical industry [1,2]. The output in terms of new drugs approved has not increased correspondingly. Although the past couple of years have shown an increase in the number of approvals [3], the viability of the current business model has been questioned and the return on investment in pharmaceutical R&D is noted to be on rapid decline [4,5]. The attrition rates of development projects and the average costs for each launched product are indeed discouraging [6]. Studies have estimated the cost of development at $1.4 billion (excluding capital costs) and $2.6 billion (including capital costs) [2, 7].

Given the huge costs involved and the deterring attrition rates, even a relatively minor productivity improvement would lead to vast savings and revenue gains [8]. It has been claimed that pharmaceutical industry R&D offers more room for productivity gains than any other research project [9]. This article is an attempt to contribute to such a productivity improvement, proposing a framework for modelling and simulation with a potential to enhance the decision-making process. We emphasise the role of uncertainty, and acknowledge the complexities intrinsic to pharmaceutical industry decision-making, which is always based on highly uncertain information [10]. Fleming [9] even claims that “ignoring the stochastic nature of drug development has led to costly mistakes and, ultimately, the industry’s decline”.

Comprehensive accounts of quantitative aspects of decision-making in pharmaceutical R&D are given by Chuang-Stein and Kirby [11] and Antonijevic [12]. Other authors have suggested procedures to optimise specific aspects of drug development programs, applying decision theoretic approaches [13, 14]. In those approaches, pre-specified utility functions are typically optimised over a limited set of parameters. We take a slightly different approach with our methodology. The methodology described here is not and attempt at finding the absolute optimum of a utility function based on restricted set of parameters. Instead, we propose to build a model flexible enough to work with a variety of situations and allow the calculation of any metric of interest based on simulation results. By repeatedly conducting simulations and evaluate the options at hand, development teams are encouraged to engage in a continuous cycle of creativity and analysis, leading to new and better investment options. The emphasis on team engagement and creation of viable alternatives is in line with the decision analysis process proposed by Nixon and Ireland [15].

Our modelling approach is based on two pillars which we think are essential for improved insight and decision-making:

- Embrace all relevant uncertainty
- Adapt an integrated holistic view of the development program and product lifecycle

We will in the next chapter elaborate further on the importance of these two pillars. The remainder of the article will introduce a framework for modelling and simulation developed to enable the enhanced decision support. A key component of this framework is a concept referred to as the ‘clinical effect model’ which will be described in some detail. The application of the methodology is then illustrated using a real-world example.

## Two pillars of a realistic modelling approach

### Embrace relevant uncertainty

Virtually all aspects affecting the success and progress of a drug development project are subject to uncertainty. For example, there is uncertainty in the time it would take to perform clinical trials, related to varying recruitment rates. There is also uncertainty in the costs involved, uncertainty in the effect of placebo, uncertainty in the existence of side effects, uncertainty in competition to market, uncertainty in the eventual sales, etc.

However, our experience is that too much of planning and decision-making have traditionally been based on fixed estimates. These estimates, or guesses, tend most often to be far too optimistic. The time it will take to finalise a trial and the costs incurred are typically underestimated. Among the many sources of uncertainty, there is one that is arguably more important, but typically neglected: The actual treatment effect of the compound under development. Many projects are planned and conducted based on a target product profile, TPP, in which an anticipated treatment effect is specified. Our experience is that the treatment effect observed will often be a disappointment compared to the numbers specified in the TPP, hence the risk of failure is often underestimated. It is a well-known fact that the vast majority of projects entering clinical development will ultimately fail. The highest rate of attrition is observed in Phase 2, and the most common cause of failure is inferior efficacy [16]. As corroborated by the degree of efficacy-based attrition, the treatment effects anticipated in TPPs may often be overestimated and the corresponding uncertainty is not adequately accounted for in design and decision-making.

We claim that adequate planning and decision-making need to account for the actual uncertainty in all relevant aspects of the development program, including the treatment effect. In doing so, we get a more realistic basis for the decisions taken and will be able to choose the program option bringing most value under realistic expectations.

### Adapt an integrated holistic approach

Traditionally, many components in a development program are designed to optimise properties locally. The determination of sample size in clinical trials is a typical example. The sample size is often calculated, based on an anticipated treatment effect, to obtain a desired power to achieve statistical significance. This sample size might be the “correct” one within the confines of the actual trial, in the sense that the desired power and significance is achieved for this particular trial (given that the assumptions are correct), However, an entirely different sample size might be optimal in order to maximise the value of the entire development program.

More generally, any decisions and design choices made in a development program will impact the remainder of the life-cycle of the product. The design and outcome from an early phase trial will impact the likelihood of successful outcomes in later phase trials. Importantly it will also impact the market success of the drug, should it eventually be launched. A couple of examples:

- A program designed to be fast in order to reach market early may lead to increased value through more years on the market with patent exclusivity. On the other hand, a program designed to prioritize speed may often stand a higher risk of failure and may be less efficient in bringing the right drug candidates to launch.
- Planning for conservative stop-go criteria in early development will lead to a lower total likelihood of approval as more drug candidate projects are terminated. However, the strict criteria may also save cost, as futile projects are terminated early. Such criteria may also imply that when a drug is eventually entering the market, there is stronger evidence that this is a highly efficacious drug.

In the examples above, there are several aspects interacting dynamically, with both positive and negative drivers of project value. These aspects relate to both the development program, the registration process and the eventual outcome on the market. In order to evaluate such dynamic effects of design choices, a model that captures the entire life cycle of the drug is needed.

## A framework for modelling, simulation and evaluation

### Process model—Flow chart representation

**Fig 1:**
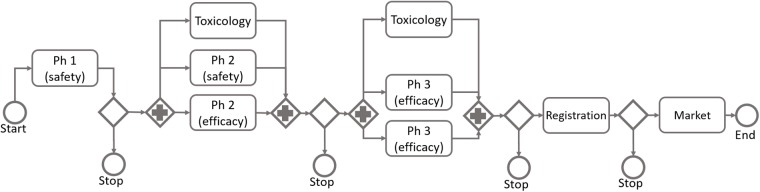
A generic example of a flow chart representation of a drug development project.

As a starting point for the modeling of a development project, we have found it useful to describe the program as a process model in a flow chart representation. We typically build these process models based on the BPMN (Business Process Model and Notation), and a simple generic example is given in Fig 1. The use of BPMN has, from our experience, proven to be a convenient way to describe and qualitatively model development programs. It is also flexible enough to cater for a vast array of situations. More details regarding BPMN can be found on the website www.bpmn.org.

Development programs include many different types of activities, i.e. toxicology studies, drug product development, manufacturing of API, etc. We recommend to include in the modelling only those activities that are considered important to inform the decisions to be supported. Based on the costs incurred, and in general the impact on decision-making, the clinical trials are often of the highest importance for inclusion in the model. Hence, we will in the remainder of this presentation focus mainly on the clinical trials, but the process model could be extended to other activities when considered of importance to the situation at hand.

### The clinical effect model

The previous chapter emphasized the need to embrace relevant uncertainty in the planning and design of development programs, and the uncertainty in terms of the unknown treatment effect was explicitly mentioned as one of the most crucial components. We describe in this section an approach taken to capture this in what we will refer to as the ‘Clinical Effect Model’, CEM.

In the CEM we assume that it is possible to identify a few endpoints that are crucial to the success of the compound, and as such central to the development program. We will introduce the concept by assuming that the evaluation is based on a single endpoint related to the efficacy of the compound. The general concept is however valid also for more general situations, e.g. the case of more than one pivotal efficacy endpoint, or endpoints related to safety. The use of the CEM in investment decisions, and its generalisation beyond the simple case of a single efficacy endpoint, are further described in a subsequent section and in the Discussion.

A fundamental part of the CEM is an assumption that each drug candidate has an inherent treatment effect that would be realised should the compound be given as a drug to the actual patient population. We will refer to this inherent property as the “true clinical effect”, *E*_*C*_. For many real-world projects, that are terminated prior to launch to the market, *E*_*C*_ is not realised as the drug will not reach the intended patient population. However, in a simulation study we can assign values to *E*_*C*_ for all projects, hence evaluating the development program based on this property. Throughout the development program, a number of trials are conducted, in which we estimate the efficacy, ${\hat{E}}_{j}$. This observed value can be viewed as representing the underlying treatment efficacy, plus a random error due to the finite number of patients included in the trial

$${\hat{E}}_{j}=E_{j}+\varepsilon_{j}.$$ (1)

A distinction is made in our model between the efficacy of the trial, *E*_*j*_, and the true clinical effect, *E*_*C*_. This difference could stem from many reasons. In earlier phases, surrogate endpoints are sometimes used to predict the eventual clinical outcome, in which case the two efficacy measures obviously differ. Also in later phases, where the study endpoint may be the same as the pivotal endpoint, features of the clinical trial may cause the efficacy of the trial to differ from the true clinical efficacy. This can be due to specific inclusion/exclusion criteria, specialized study sites differing from general hospital clinics, etc. To account for the uncertainty in the treatment effect, *E*_*C*_ and *E*_*j*_ are treated as stochastic variables to which we assign distribution assumptions when the model is evaluated. The assumptions regarding these distributions will be based on the accumulated knowledge available at the time of modelling. In late phase development there might be earlier clinical trials providing estimates, and in early phase development, translational science data may be used. Benchmark data on treatment effects and success probabilities in the targeted indication might also be used.

An important property of a study is its ability to provide accurate prediction of the true effect (i.e. *E*_*j*_ should be an accurate predictor of *E*_*C*_). A clinical trial using a biomarker in a selected subpopulation of patients may be assumed to have less predictive accuracy than a trial using the clinical endpoint in a general patient population. Hence, the performance of various drug development strategies may depend strongly on the predictive accuracy of the different trials.

The predictive accuracy is in the CEM represented by the correlation between the ‘true clinical effect’ and the ‘true efficacy in the trial’, *ρ*_*j*_ = *corr*(*E*_*j*_, *E*_*C*_). This correlation is a key feature in the CEM. It is reasonable to believe that the endpoint measured in an animal model in pre-clinical development is only moderately correlated to the true clinical efficacy, corresponding to a low value of *ρ*_*j*_. On the other hand, the endpoint measured in a Phase 3 clinical trial should be assumed to have a fair correlation to the true clinical efficacy, i.e. a high value of *ρ*_*j*_. Taking the predictive ability into account when evaluating various design options, i.e. by assigning a relevant value of the correlation, is essential to obtain an adequate assessment of the relative merits of the options under evaluation.

### Attributes for activities

As described in Sec 3.1, it is recommended to include in the model all activities throughout the entire development program that are of importance to inform the decision at hand. Attributes are assigned to each activity. In our model the main attributes are:

- Cost, *C*_*j*_
- Time, *T*_*j*_

The most important activities in a drug development program are often the clinical trials, for which the sample size of the trial is also a central attribute. This allows to capture the typical situation that the cost and time of a trial would be a function of the number of patients included. We define *N*_*j*_ as the number of patients per treatment arm and will introduce the methodology assuming that there is equal allocation to the treatment arms.

For this example we chose a simple model where the cost is proportional to the sample size plus a fixed cost for the trial. If the cost per patient is $C_{j}^{N}$ and the number of treatment arms is *A*_*j*_, the cost of the trial is

$$C_{j}=C_{j}^{0}+A_{j}N_{j}C_{j}^{N}$$ (2)

The time of a trial would also be a function of the sample size. We choose a model where the time is proportional to the sample size plus an additive component. If the recruitment rate is *R*_*j*_ patients per year, the time of the trial is given by

$$T_{j}=T_{j}^{0}+A_{j}N_{j}/R_{j}$$ (3)

The examples given above are relatively simple models to capture a couple of the most essential properties of a trial. An increase in sample size comes at a cost, both in financial terms and in calendar time. The model could of course be made more elaborate, whenever the decision to be supported warrants increased detail. One example is to include the number of centres in the trial. The cost is then defined as an increasing function of the number of centres, and the time defined as a decreasing function.

### Investment decisions and the clinical effect model

In a development program, there are typically a number of decision points, where choices are made on how to proceed with the project, e.g. whether to stop the development of the compound or to invest in the next phase of development. These decisions points (e.g. stop/go or Go/No-go decisions) are important elements of our models. One simple way of modelling the attrition occurring at the decision points is to assign a fixed probability of a successful progression to the next phase. However, a more realistic approach would be to model the investment decision as dependent on the observed efficacy of the key trials in the previous development phase. Consequently, in our model the investment decisions are linked to the CEM.

The criteria for progressing to the next phase are often based on the statistical significance of the results observed. Using this approach, a positive investment decision is made if the p-value from a previous trial is lower than a given threshold, ${\hat{p}}_{j}<p_{j}^{crit}$. Alternatively, the criterion could be defined as a test statistic, *z*, exceeding a given threshold, ${\hat{z}}_{j}>z_{j}^{crit}$. As an example of a simple model, we may assume that the investment decision is related to the comparison of the treatment effect of two treatment arms, and that the test statistic underlying the decision can be approximated by the comparison of two treatment means (cf the standard two-group t-test). With ${\hat{E}}_{j}={\bar{X}}_{active}-{\bar{X}}_{placebo}$, we will in this case have

$${\hat{z}}_{j}=\hat{E_{j}}/SE({\hat{E}}_{j})=\frac{{\hat{E}}_{j}}{s\sqrt{2/N}}$$ (4)

where s is the standard deviation on the endpoint in question, and SE is the corresponding standard error of the difference in means. We have in this section illustrated the case of decision criteria based on statistical significance of efficacy. In the Discussion we will give some examples on how other, more complex, decision criteria can be modelled within the framework.

### The development program as a selection process

**Fig 2:**
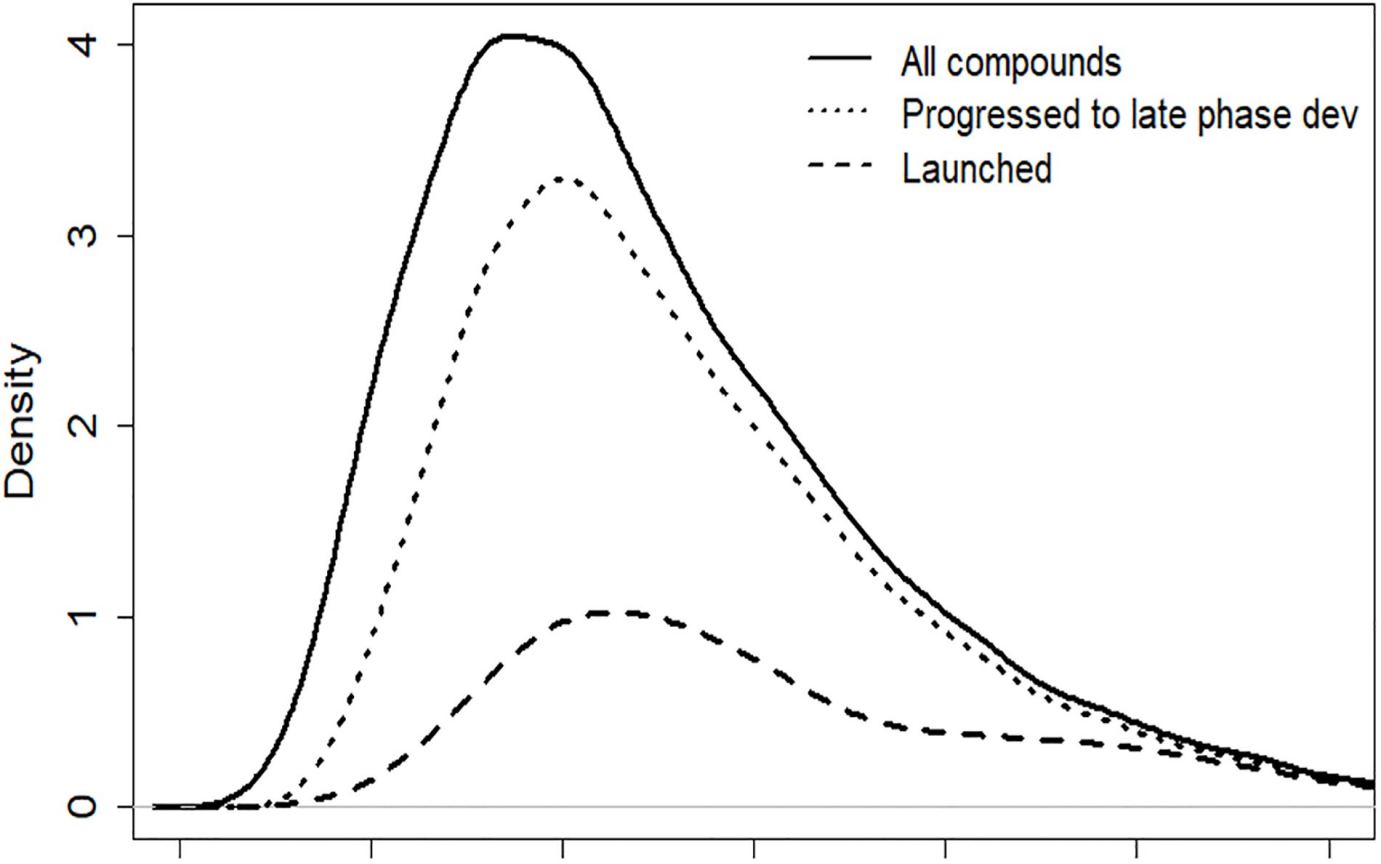
An example of a distribution of the true effect for subsets of simulated compounds progressing through development. (The horizontal axis is an arbitrary scale of treatment effect. The density functions have been scaled proportional to the proportion of compounds remaining after each phase).

Stop/go criteria as outlined above will contribute to attrition and gradually weed out compounds showing futile efficacy. Compounds with a desirable efficacy stand a better chance of being progressed to later development phases. Consequently, it should be the case that compounds at the end of a development program will (on average) have a better efficacy than those entering the first phase of development. However, due to the random component of measurement errors, and since the efficacy measured in trials is not identical to the true clinical effect, the selection will not be perfect. This is illustrated in Fig 2, where simulation results from a Clinical Effect Model are shown. The black curve represents the distribution of the true treatment effect for compounds entering clinical development. As compounds are selected through the early development stop/go criteria of investment decisions, a proportion of the left-hand part of the distribution is removed. The result is a shift of the remaining distribution (dotted line) partly to the right. In late development there is further attrition and the dashed line shows the distribution of compounds actually launched. The graph illustrates the fact that compounds with superior efficacy have a relatively larger chance of being retained throughout the program. However, from the graph it is also obvious that a substantial part of compounds with a relatively high efficacy will be subject to attrition (‘false negatives’). There are also cases in which compounds with inferior efficacy are retained throughout the entire program (‘false positives’).

### The clinical effect model and the probability of success

In a traditional approach, the probability of success, PoS, is given as a fixed probability, e.g. based on a benchmark assumption or taken as the power for which a study is designed. In our approach, the PoS is typically not an assumption but an outcome from simulations of the model. Several parameters of the model will interact dynamically, resulting in an observed PoS. Some of these parameters are:

- Level of statistical significance to declare success
- Required observed efficacy to declare success
- Assumptions on the true efficacy of the compound
- Sample size of the study
- Standard deviation on the endpoint of interest

In addition, the PoS of later phase trials will be dependent on parameters pertaining to preceding studies and investment decisions in the development program. This is due to the fact that the preceding investment decisions will constitute a selection process, resulting in a change in the distribution going into the next phase of development (see Fig 2). Hence, the distribution driving the outcome of a later phase trial will be different from the distribution that we might assume at the point of modelling.

### Sales revenue and the clinical effect

An important part of the CEM is appreciating the fact that a drug shown to have a very good treatment effect is likely to generate more revenue than a drug with a mediocre effect. A relevant model of a drug development program should therefore include a dependency between treatment effect and sales. We will here introduce a simple way to model such a dependency.

In a basic commercial forecast, the sales revenue can be assumed to follow a trapezoidal curve over time. Following registration and launch there will be a period of ramp-up sales, followed by a plateau where a peak sales (PYS) is reached. As the drug reaches the time for loss of exclusivity on the market (LoE), sales will drop to a much smaller volume (residual sales). Formally, we can write a model for the sales at time *t* as:

$$Sales_{t}=\{\begin{array}{ll} \;\frac{\left(t-T_{L}\right)}{U}*PYS & if\;T_{L}<t\leq T_{L}+U\\ PYS & \;if\;T_{L}+U<t\leq T_{E}\\ f*PYS & \;if\;T_{E}<t \end{array}$$ (5)

where *T*_*L*_ is the time of launch, *U* is the length of the sales ramp up period, *T*_*E*_ is the time of LoE and *f* is the proportion of sales remaining after LoE.

It is reasonable to assume that the observed treatment effect in Phase 3 trials, ${Ê}_{3}$, will (at least initially) affect the sales. Assume that *Sales*_*t*_ is the anticipated revenue under the assumption that the treatment effect is the one specified in the TPP, denoted *E*_0_. A simple model to accommodate for effect dependent sales might then be

$${Sales}_{t}^{E}={Sales}_{t}*\frac{{Ê}_{3}}{E_{0}}$$ (6)

The sales, at time *t*, is here modelled to be proportional to the observed Phase 3 results, ${Ê}_{3}$ calibrated with the TPP-based treatment effect, *E*_0_, and the corresponding sales forecast, *Sales*_*t*_.

The impact of the treatment effect on sales can of course also be modelled in several other ways. Should one assume that many other sources of knowledge are available, and that the market would soon know the true clinical effect of the drug, one might replace ${Ê}_{3}$ in Eq 6 by the true clinical effect, *E*_*C*_. A further alternative might be to decompose sales into volume and price components. The treatment effect can then be modelled to affect either of the volume or the price (or both).

### Simulations and performance indicators

Once the model has been defined as described in previous sections of this chapter, we use computer simulations to evaluate properties of the development program. A prominent feature of the proposed modelling approach is that all relevant sources of uncertainty should be explicitly embraced. Consequently, when conducting simulations, model parameters are typically not given a fixed value, but for many parameters we assume a stochastic distribution to reflect uncertainty.

As an example, the true effect of a drug candidate, *E*_*C*_ and *E*_*j*_, might be drawn from a lognormal distribution. Other attributes that would typically be represented with uncertainty, hence drawn from a stochastic distribution when performing simulations, are the time and the cost of the trial, and the parameters driving time/cost as defined earlier. The simulation will then in each iteration draw random numbers from the assigned distributions.

From the simulations, any number of performance indicators can be calculated. The following list includes just a few examples which from our experience have been frequently useful:

- Probability of Launch
- Expected Net Present Value, eNPV
- Expected gain, for the subset of iterations proceeding to successful launch
- Expected loss, for the subset of iterations terminated prior to successful launch

Based on these measures, and any other indicators that may be considered useful, development options can be evaluated and compared. It will also typically prove very useful to present visual graphics, gaining further insight and supporting decisions to be made. Some examples will be given in the empirical application illustrated in a subsequent chapter.

### Financial metrics and cash flow models

Several parts of our model represent financial events, with typical examples being the cost incurred by clinical trials and the sales revenue gained once the product is launched into the market. As a result, various financial metrics could be calculated from the model and used to describe and evaluate properties of a development program. The return on investment, ROI [17], and the net present value, NPV [18], are typical examples. In the literature on decision-making in the pharmaceutical industry, the NPV seems to be the primary metric of choice [13, 18–21]. I will follow this example and use NPV for illustration, while noting that other metrics may also be informative and could easily be calculated based on the proposed framework.

**Fig 3:**
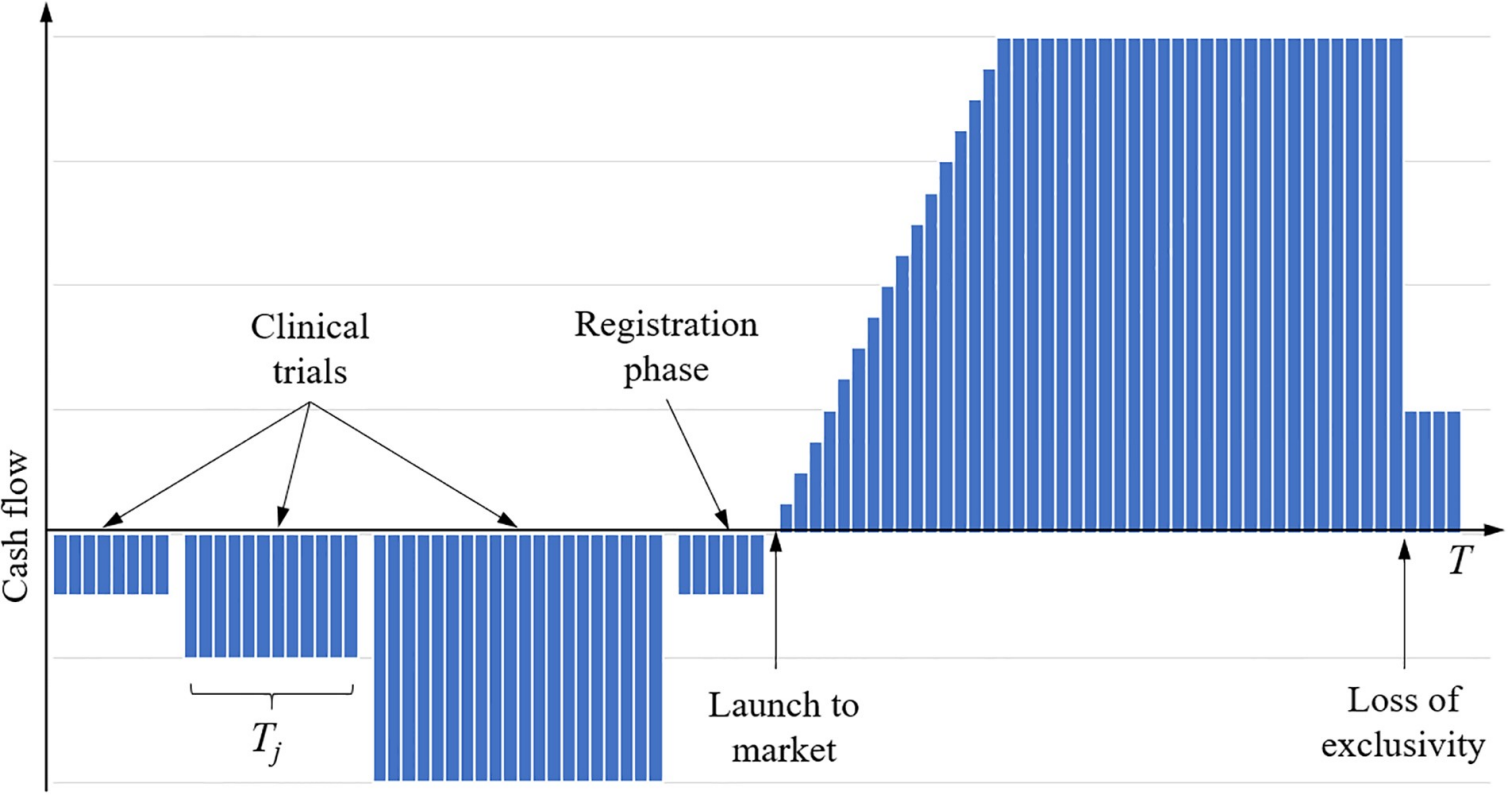
Schematic illustration of the cash flow profile from a development program.

The calculation of NPV is based on obtaining the net cash flow during the evaluation period. We have chosen to implement a discrete model, where the cash flows are attributed to a finite number of time buckets. Let the time period of interest be *t* ∈ [0, *T*], and let the length of each time bucket be *τ*, implying a total of *I* = *T*/*τ* time buckets. Assuming that the costs, *C*_*j*_, from a clinical trial (or more generally any activity in the model) are approximated to occur at a constant rate over the duration of the trial, *T*_*j*_, we have that the cash flow incurred by the trial at time bucket *i* is $F_{ij}=\frac{-C_{j}}{T_{j}/\tau }$. Similarly, the sales revenue for the market phase is transformed into a cash flow for each time bucket after launch by discretising the sales model (cf Eqs 5 and 6). For the general case that more than one activity may be run in parallel, the resulting cash flow at a time bucket is *F*_*i*_ = Σ_*j*_ *F*_*ij*_. A schematic illustration of the cash flow profile from a development program is provided in Fig 3.

With the cash flow, *F*_*i*_, defined for the entire time period of interest, the NPV is calculated by discounting the cash flows as

$${NPV}_{k}=\sum_{i=1}^{I}F_{i}{\left(1+r\right)}^{-t_{i}}$$ (7)

The index *k* is used here to represent a specific iteration of the simulation. The aggregated metric of interest will then be the expected NPV, as given by the average

$$eNPV=\frac{1}{K}\sum_{k=1}^{K}{NPV}_{k}$$ (8)

### Algorithm for implementing the proposed framework

The proposed framework for modelling and decision-support is a general methodology intended to be applied to a wide variety of situations and development programs. While the details will be different for each situation, this section summarizes in a general stepwise algorithm the implementation of the methodology. For some steps in the algorithm, references are given to earlier sections of this chapter where details are given.

1. Choose meta-parameters for the model and analysis:
  - Set the number of simulation runs, *K*
  - Set the length of the calculation horizon, *T*, and the length of time buckets for cash flow calculations, *τ* (cf “Financial metrics …”section)
  - Set the discount rate, *r* (cf “Financial metrics …”section)
2. Choose what activities and decision points to include in the model:
  - It is recommended to illustrate the modelled development program as a process model, e.g. using BPMN (cf “Process model …”section)
3. Define attributes for activities (e.g. clinical trials):
  - Choose how to model the cost of an activity, e.g. as a fixed value or as a function of sample size and/or number of study sites (cf “Attributes …”section, Eq 2)
  - Choose how to model the duration of an activity, e.g. as a fixed value or as a function of sample size and/or number of study sites (cf “Attributes …”section, Eq 3)
  - Repeat *(a)* and *(b)* for each activity in the model
4. Define success criteria for decision points:
  - Choose the success criteria for a decision point (cf “Investment decisions …”section)
  - Repeat *(a)* for each decision point in the model
5. Generate simulated data for input parameters (cf “Simulations …”section):
  - Draw values from a stochastic distribution (for all input parameters where uncertainty is modelled)
  - Set to fixed values (for input parameters without assigned uncertainty)
6. Evaluate a simulation iteration:
  - Calculate attributes, i.e. cost and duration, for each activity (as defined in step 3)
  - Evaluate outcomes of the decision criteria for each decision point (as defined in step 4)
  - Register the path through the development process, resulting from the decision point outcomes
  - Register what activities are performed in this path of development
  - Register the cost and duration for these activities
  - Calculate the time at which the activities occur. Note that this time may be subject to random variation, as the duration of previous activities may be modelled with uncertainty
  - Calculate the sales revenue time series, given that the development path obtained in *(c)* did not lead to a project termination prior to launch (cf “Sales revenue …”section)
7. Calculate cash flows (cf “Financial metrics …”section):
  - Calculate a net cash flow for each time bucket, based on the cost and revenues obtained in Step 6
  - Perform a discounting of the cash flows using the chosen discount rate, and calculate metrics of interest, e.g. NPV (Eq 7)
8. Repeat steps 5–7 for each of the *K* simulation iterations
9. Perform analysis of the obtained results:
  - Calculate aggregate performance metrics of interest (cf “Simulations …”section and Eq 8)
  - Produce output visualizations of output results

## An illustrated example

### The project and the program design

**Fig 4:**
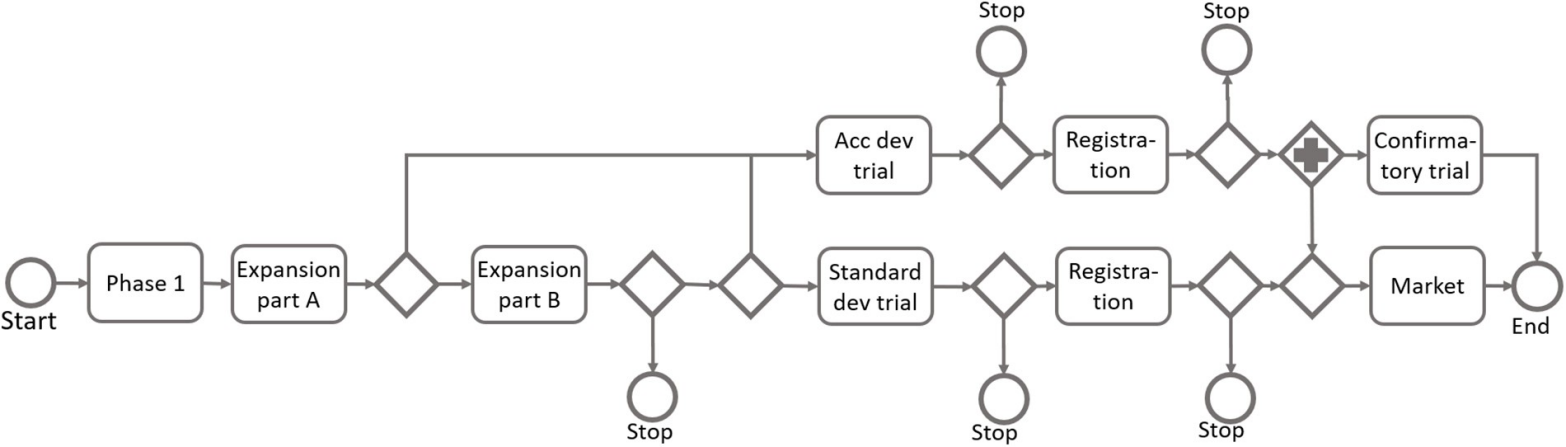
Process model of the development program used for illustration.

In this chapter we will illustrate the application of the proposed modelling approach by means of a worked example. The example is based on a real-world oncology project, but anonymized and slightly modified. The development program is illustrated in a process model in Fig 4.

The Phase 1 trial is planned to be continued in a so-called expansion trial, where additional cohorts of patients are included, and in which efficacy is evaluated. There are potentially two expansion cohorts. After the first expansion cohort there is a possibility to go directly to an accelerated development path, provided that the results are exceptionally promising. Less exceptional results would lead to a second expansion cohort. After the second cohort there is an investment decision, at which the project is either terminated, continued to the accelerated development path, or continued to a standard development path. The accelerated path is based on overall response rate as the endpoint. This is expected to be sufficient for registration for this indication if results are sufficiently promising. The standard development path includes a randomized controlled trial, with progression free survival as the primary endpoint. A confirmatory trial is also expected after an approval from the accelerated path.

### Model attributes, simulation and summary of results

For the activities and decision points outlined in the process model, we have assigned assumptions to parameters as defined in the model description of the previous chapter. Details on the assigned parameter values are provided as supplementary material. The modelling and simulations for this example were performed using a purpose-built tool, Captario SUM, developed specifically to facilitate application of the proposed methodology in drug development projects and portfolios.

**Fig 5:**
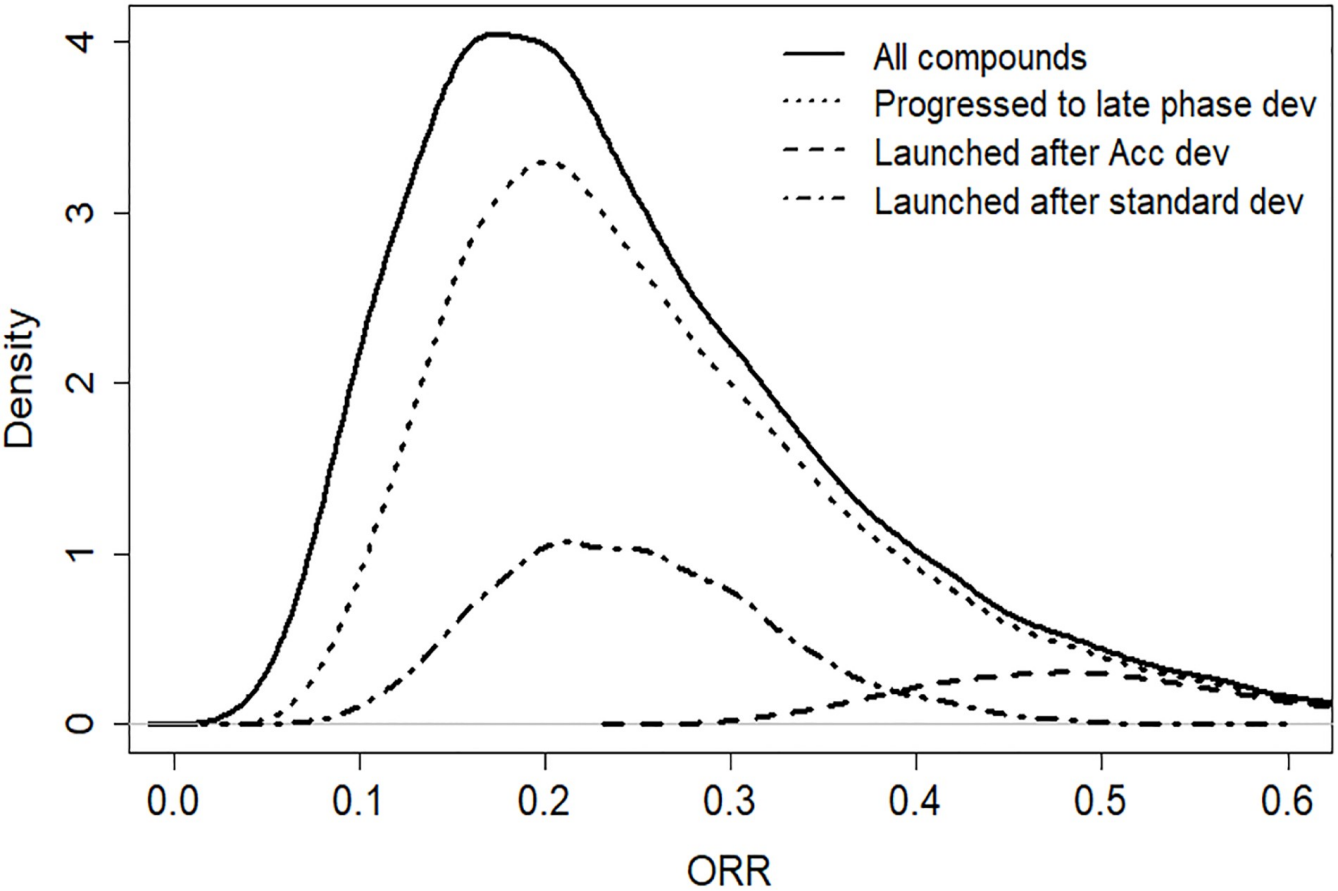
The distribution of the true effect on overall response rate. Distributions are given for all iterations, as well as for subsets of iterations progressing through the different paths of development. (The density functions have been scaled proportional to the proportion of iterations in each case).

A Monte-Carlo simulation was then run with 10000 iterations. This would correspond to 10000 potential outcomes of the development program, reflecting the uncertainty and dynamics of the large number of parameters of the model. Each iteration would represent a draw from the assumed distribution for the true effect, illustrated by the solid line in Fig 5. The dotted line represents the distribution of compounds successfully progressed after the expansion trial. The dashed line distribution shows that compounds reaching launch after being progressed through the accelerated path will generally have a very high true effect on the ORR scale. On the other hand, the dashed-dotted distribution indicates that compounds launched after the standard development path, will in this case typically have a moderate treatment effect. These results illustrate the outcome for the current example of the selection process that is obtained through the Stop/Go decisions of drug development programs.

We mentioned in an earlier section a few examples of summary metrics that is often of interest. For the illustrated example these measures were estimated at:

- Probability of Launch           31%
- Expected Net Present Value, eNPV 45 MUSD
- Expected gain              236 MUSD
- Expected loss              40 MUSD

### Decision support based on the model

In this section we will illustrate how the model can be used to support some strategic decisions relevant to this project.

- Choice of decision criterion for a stop/go decision after the expansion trial
- Choice of sample size for the randomized controlled trial
- Assessment of capital requirements over time

### Choice of decision criterion

Referring to the process diagram of Fig 4, we can see that there is a decision point after the expansion trial, at which there is a choice to be made whether to stop the program, to continue with an accelerated development path or to continue with a standard development trial. The decision on which path to take will have a substantial impact on the project, and the choice of decision criteria is consequently important. The primary endpoint of the expansion trial is the overall response rate (ORR). The stop/go criterion is based on the number of responders required, among the 40 patients (20 patients in each of the two expansion cohorts), to proceed the project.

**Fig 6:**
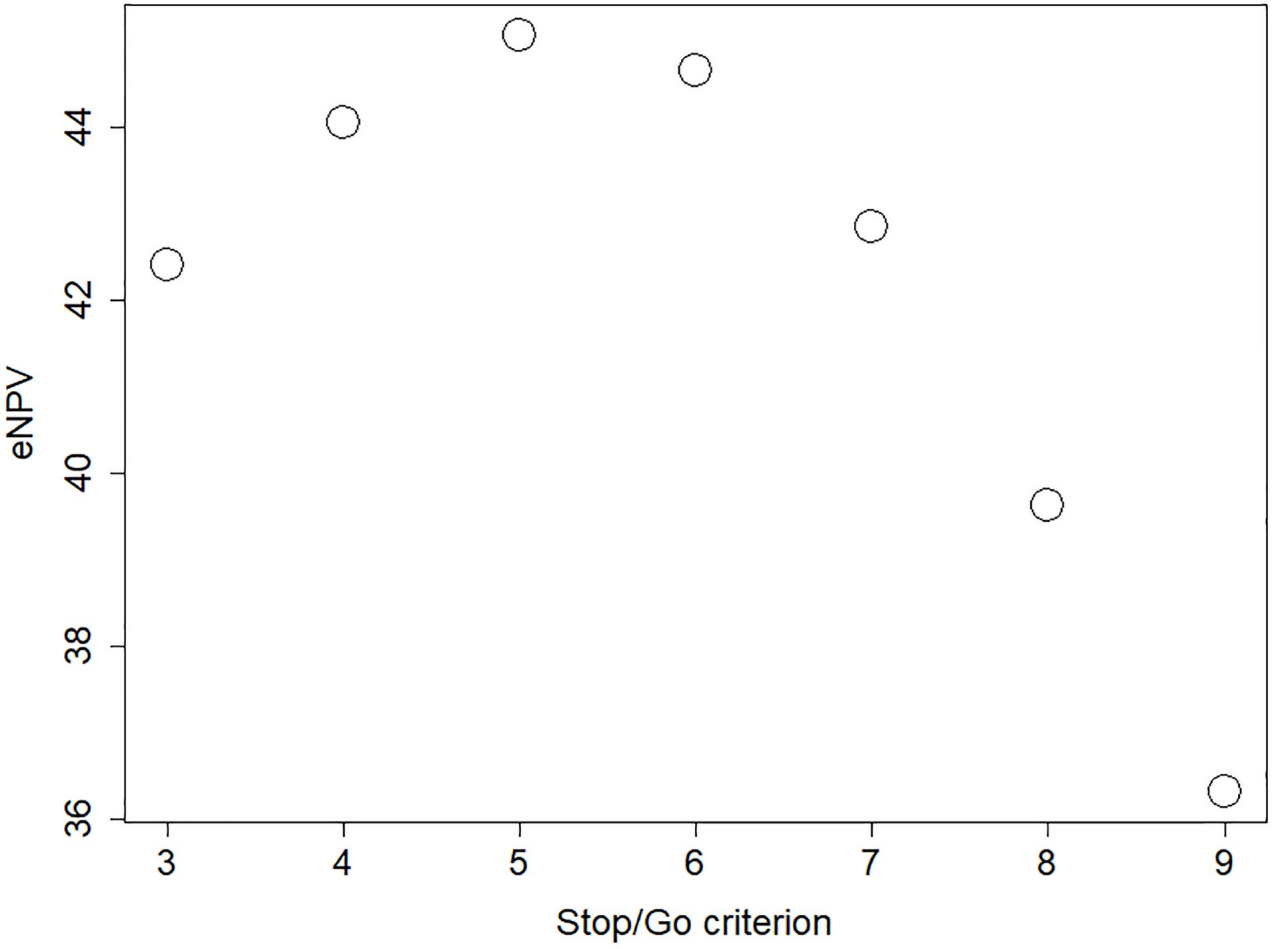
The mean net present value for a range of stop/go criteria applied after the expansion trial.

A criterion initially considered by the project team was to require an ORR of at least 20%, i.e. at least eight responders. To support this choice, a simulation was run where the decision criterion, $E_{j}^{crit}$, varied over a range of values. Fig 6 shows the expected NPV for a range of values on the decision threshold, and it illustrates that the initially suggested decision criterion, $E_{j}^{crit}=8$, is probably too conservative. A substantially higher project value would be obtained with a more liberal criterion, e.g. $E_{j}^{crit}=5$, and results indicate that this choice of decision criterion would increase the project value with approximately 13%.

### Choice of sample size

The choice of appropriate sample size for clinical trials is an issue pertinent to any drug development program. In this example, we illustrate the proposed modelling approach on the assessment of sample size for the randomized controlled trial in the standard development path.

**Fig 7:**
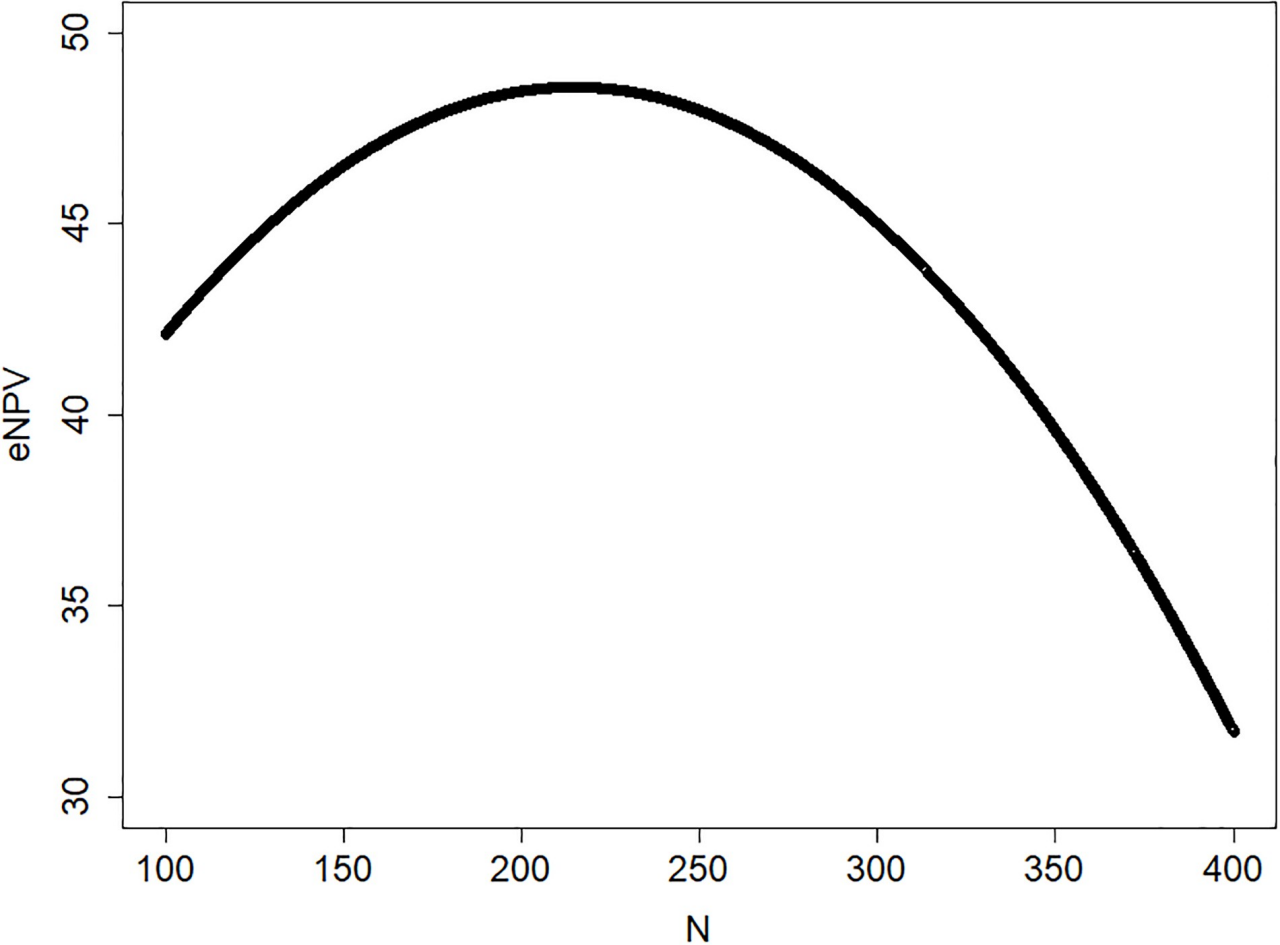
The expected net present value as a function of the sample size of the randomized standard development trial.

The provisional sample size suggested by the project was N = 300 patients per treatment arm. When running simulations on the model, we let the sample size vary on the range between N = 100 and N = 400, and for each iteration, the NPV was calculated. These data allow to fit a function to the relation between NPV and N (Fig 7). The results indicate in this case that the value of the project is larger for a somewhat smaller sample size than proposed by the project team, with an optimum at N = 220.

### Assessment of capital requirement

The previous two paragraphs illustrated the use of our methodology to support decisions on program design choices. Another use of the model is to assess operational aspects of the development program. As described earlier, each activity (trial) is in the model given a number of attributes, e.g. time and cost. Typically, these attributes are given with relevant uncertainty.

**Fig 8:**
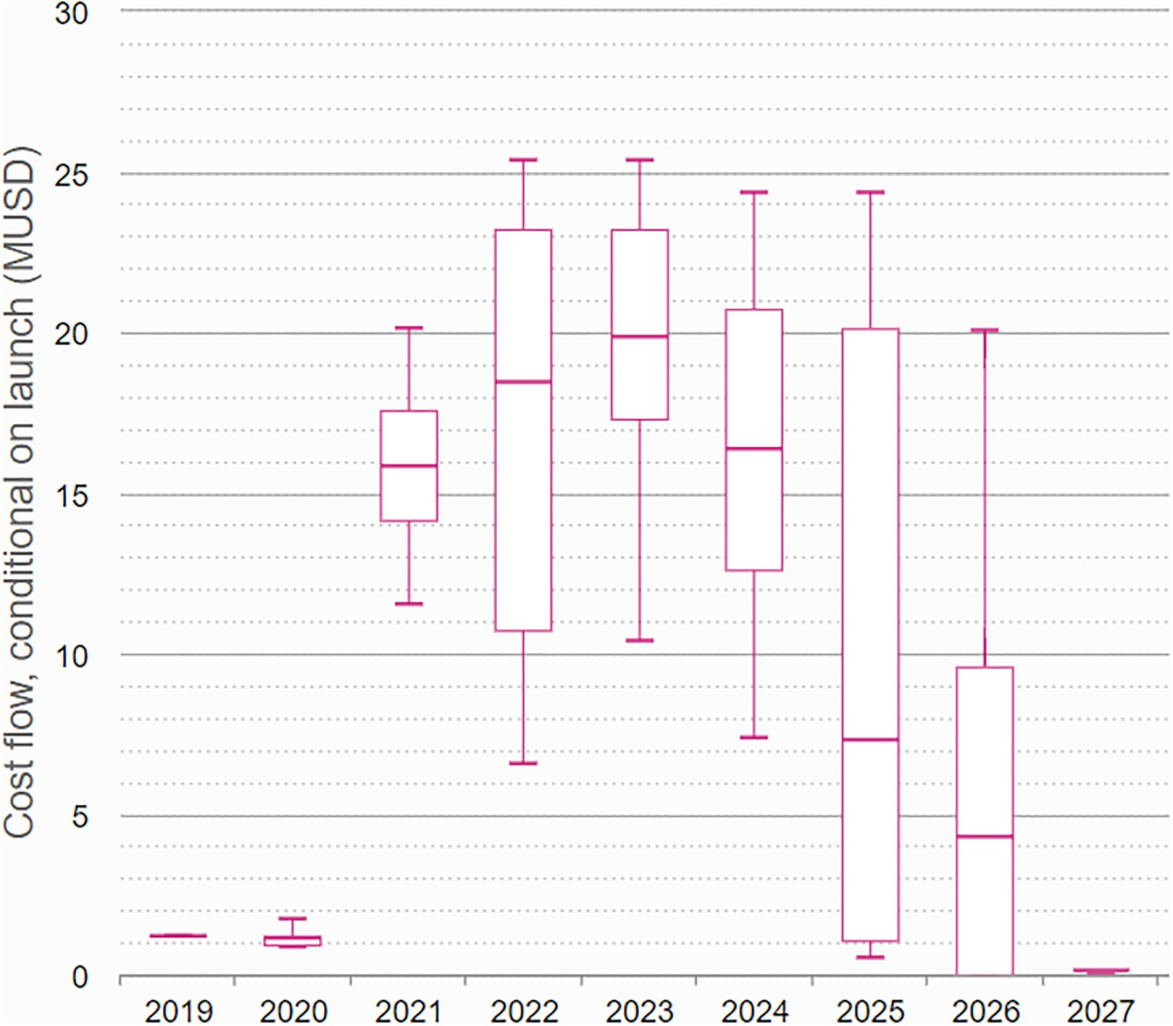
The cost incurred throughout the development program, illustrated by box plots over time, for the subset of simulation iterations leading to a launch.

**Fig 9:**
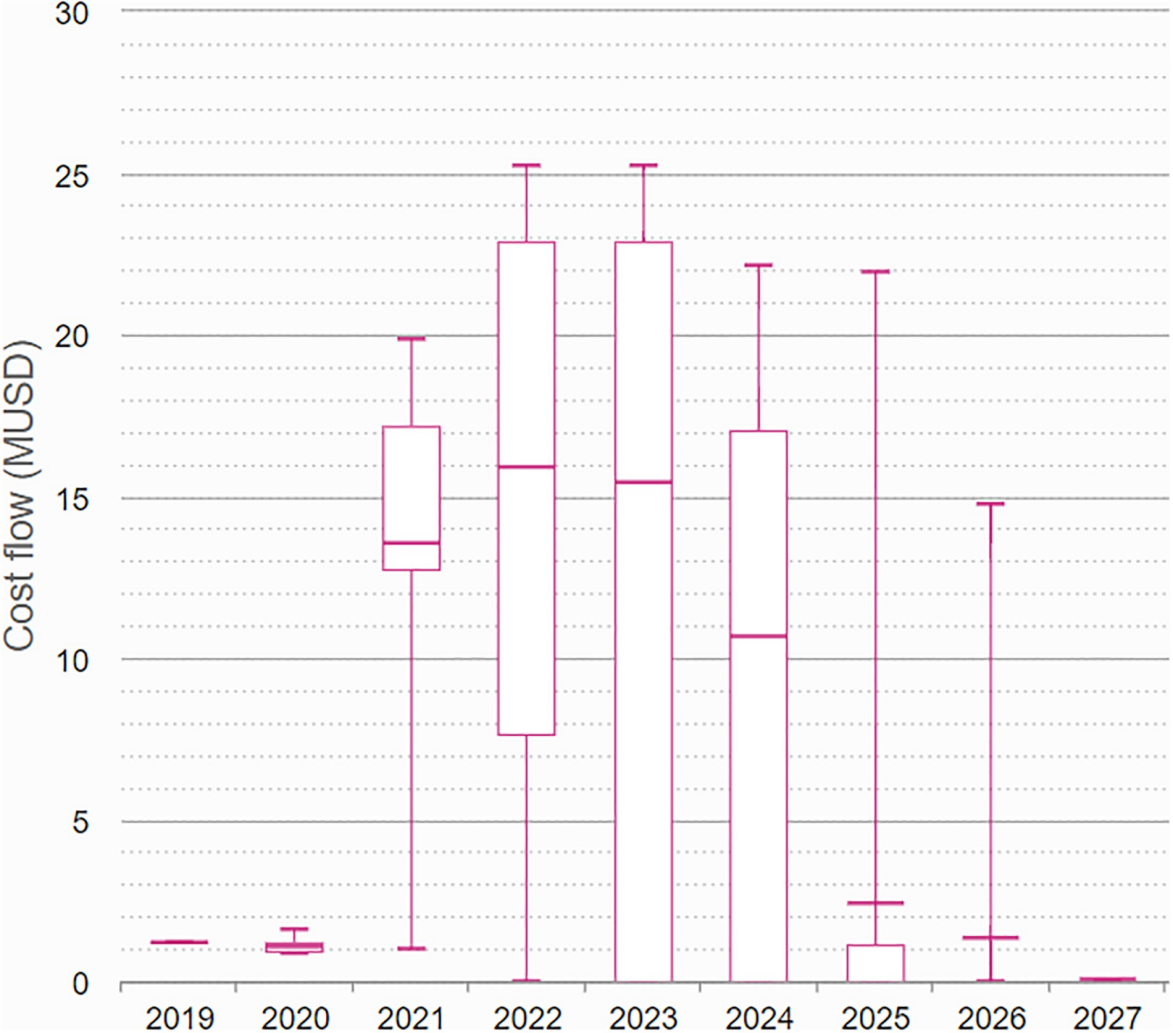
The cost incurred throughout the development program, illustrated by box plots over time.

Several operational properties of the development program can then be derived as an outcome of the simulations. In Figs 8 and 9 we illustrate the development cost incurred by the development program, showing how the costs develop over time. Fig 8 shows the cost for the subset of iteration where the project leads to a launch, whereas Fig 9 shows the cost for all iterations of the simulation. The box plots are useful in assessing the budgetary requirements for the project, both in terms of the monetary value and in terms of their timing. The box plots also illustrate clearly the large degree of uncertainty in the costs drawn by the project.

## Discussion

### The illustrating example

The methodology proposed in this paper is designed to be flexible enough to allow the support of a vast variety of decisions and design choices throughout a development program. The case study in the previous chapter illustrates just a few examples of the type of decisions and assessments that could be supported:

- Choice of stop/go criteria at decision points
- Clinical trial design issues
- Assessing capital requirements and other financial aspects of a development program

The results regarding the choice of decision criteria are the outcome of a rather complex interplay between several factors. A high decision threshold would lead to a reduced risk of progressing a futile drug candidate (i.e. fewer false positives) and it would thereby reduce development costs in later phases. A high threshold would also imply a strict selection so that, referring to Fig 5, the distribution of the true effect of the progressed drug candidates would be shifted towards higher treatment effect. On the other hand, a high threshold would lead to a termination of the project in many cases where the true effect was actually acceptable (i.e. a large number of false negatives), and these would correspond to missed opportunities of revenue from launched product. In addition, the balance of the mentioned aspects will also depend on the program design and decision criteria of the later phases. Our modelling approach and the corresponding simulations will, in these complex situations, reveal dynamics and lead to insights that would not have been possible to reach with traditional calculations. It may be noted that the results obtained in this case study are in line with findings in Miller & Burman [14], where they show some theoretical background to why more liberal decision criteria are in some situations beneficial in early phase decision-making.

The issue of determining an appropriate sample size is present in any drug development project, and in almost any clinical trial. The sample size has traditionally implied a calculation exercise for the statistician. Our modelling approach enables a different view on how to inform the choice of sample size. Firstly, the true treatment effect of the drug candidate is never known, and it may be argued that an appropriate sample size calculation should take this uncertainty into account. The concept of assurance [22] is to some extent addressing this issue, in that it involves a prior distribution to be set on the treatment effect. Secondly, the obsession with the 5% significance level is unfortunate, and other levels would often be beneficial. There are many situations where a higher (or lower) significance level would be appropriate [14]. Thirdly, the statistical power as calculated on a single study is not necessarily a useful metric on which to base strategic decisions. We argue that important decisions should be made based on a metric capturing wider aspects of the development program. Such metrics could be global economic measures, e.g. NPV or ROI, or components contributing to the project value, like the probability of launch, the expected loss if project fails, the expected gain if project succeeds, etc.

In our third example, we derive the development costs for the drug project. In contrast to traditional forecasts, these results do represent the outcome when relevant uncertainties are taken into account. In any development program, the exact amount and timing of costs to be incurred would not be known in advance, and anyone with a background in the pharmaceutical industry will have seen both delays and cost increases in their development projects. Appropriate budget planning should incorporate these uncertainties to ensure an acceptable probability that the budgetary limits will hold. In particular for small biotech companies, an appropriate forecast of future capital requirements is essential to plan when venture capital injections are needed.

### Generalizing the decision criteria

The Clinical Effect Model, CEM, and its relation to investment decisions was introduced in Sec 3.2 and 3.4 using the statistical significance of an efficacy endpoint as the decision criterion, i.e. a positive decision is concluded if ${\hat{z}}_{j}>z_{j}^{crit}$. However, the situation is sometimes more complex, including more than one parameter, and I will here discuss some examples on how the methodology can be generalized to model other types of decision criteria.

- *Statistical significance and clinical relevance*, ${\hat{z}}_{j}>z_{j}^{crit}$ and ${\hat{E}}_{j}>E_{j}^{crit}$.
  It is often stated that a positive outcome should require, in addition to statistical significance, also a certain size of the treatment effect. As noted by Saint-Hilary et al [23], these two criteria may often, for a given set of design parameters, be redundant. However, keeping both rules in the criteria may permit changing other parts of the model and performing analysis without changing the decision criteria.
- *Statistical significance and absence of critical safety signal*, ${\hat{z}}_{j}>z_{j}^{crit}$ and *I*_*j*_ = 0,
  where *I*_*j*_~*Bernouilli*(*p*_*j*_) may be an indicator for the occurrence of an idiosyncratic safety signal, which occurs independent of the efficacy endpoint.
- *Clinical relevance and acceptable level of a safety parameter*, ${\hat{E}}_{j}>E_{j}^{crit}$ and ${\hat{S}}_{j}<S_{j}^{crit}$
  The clinical efficacy and safety parameters may, in this case, be assumed to be correlated. The effect modelling approach can be applied also to the safety parameter, generating ${\hat{S}}_{j}$ as described earlier for ${\hat{E}}_{j}$ (cf Eq 1), and ensuring appropriate correlation, $\rho_{j}^{S}=corr(S_{j},E_{C})$, between the safety parameter and the clinical efficacy. As an example, we have applied this type of criterion for models in stroke prevention, where the primary efficacy (prevention of stroke events) is likely to be negatively correlated to a known safety issue (occurrence of bleeding events).
- *Statistical significance*, *clinical relevance and positive benefit-risk*, ${\hat{z}}_{j}>z_{j}^{crit},{\hat{E}}_{j}>E_{j}^{crit}$ and $\Delta {\hat{U}}_{j}>0$
  Saint-Hilary et al [23] propose to use a composite measure as the basis for definition of success, including the three above-mentioned components. The essence of the benefit risk component is to allow for a comparison between the investigational treatment and control, by combining information on both efficacy and safety parameters. A brief outline of how the main ideas could be implemented in our framework is as follows. Assume that there are *L* safety parameters of particular interest. For each parameter, the difference between treatment groups is transformed to a value on the interval [−1,1], with 0 representing treatment equality. We then have the estimates ${\hat{E}}_{j}^{*}$ and ${\hat{S}}_{jl}^{*}$, *l* = 1,..,*L* which can be defined and simulated in line with the general methods described earlier in “The clinical effect model” section. For each of the parameters, a weight, *w*_*l*_, is assigned such that $\sum_{l=0}^{L}w_{l}=1$, with *w*_0_ being the weight of the efficacy parameter. The composite measure can then be calculated as
  $$\Delta {\hat{U}}_{j}=w_{0}{\hat{E}}_{j}^{*}+\sum_{l=1}^{L}w_{l}{\hat{S}}_{jl}^{*}$$
  and can be used as one of the decision criteria.

While the above bullet points illustrate a few specific examples of decision criteria, it should be clear that the proposed framework is flexible enough to cater for a wide variety of situations.

### Related work and general comments

We have in this article described the proposed framework mainly from the perspective of the clinical trials leading up to registration and launch to market, while the importance of considering other activities in a development programs was mentionedbreifly. Other authors have placed the focus on other areas than the clinical trials. Related work has for instance been done by Marques et al [24–26], with a focus on manufacturing aspects in drug development. Marques et al develop methods to support decision-making related to process design and production planning during drug development.

We described earlier the calculation of financial metrics based on a discrete cash flow model, Other authors [20] have chosen a continuous cash flow representation for this type of modelling. Fig 3, schematically illustrating the cash flow during a development program, shows that the basic implementation of our model assumes that costs are distributed evenly over the duration of clinical trials. If modelling of more detailed cost allocation is required, cost allocation could be made more flexible, e.g. allocating additional costs at start and end of the trial [20]. For the market phase, Patel et al [20] chose to approximate the sales revenue as constant during the market phase, dropping to zero after patent expiry. We suggest in Eq 5 a slightly more elaborate sales model. Our model is more in line with the one used by Patel et al [19], with a sales ramp-up at the first years on market, and a residual sales following patent expiry (loss of market exclusivity).

While financial metrics, such as the eNPV, have been extensively used in many situations, it should be noted that other types of metrics could also be of value. In the early phases of development, the sales forecasts may be considered too uncertain and other non-financial measures might be of more interest. The proposed framework allows for the calculation of a wide range of summary metrics based on the simulated data. In particular, we note that the modelling of a treatment effect through the Clinical Effect Model allows for the evaluation of the ability of the development program to select the right compounds for progression to launch (c.f. Sec 3.5, Fig 2). Based on the simulation outcomes one could for instance estimate the probabilities of false negatives (projects terminated although having good efficacy) and false positives (projects launched although having mediocre true efficacy).

Mathematical modelling is essential when quantitatively evaluating the properties of a drug development program and providing supporting evidence for decisions to be made in various situations. However, the way in which models are employed may differ between applications. In some cases, when the focus is on addressing a specific question, it may prove useful to find analytical solutions to a mathematical formulation of the problem [14, 20]. The analytical solution may also allow for a formal optimization of certain parameters within the confines of the defined model. In other cases, authors have chosen to present results based on computer simulations based on underlying models [11, 19, 21]. The simulation approach has its main advantages in situations where analytical solutions are hard to find, and where flexibility to easily adapt and evaluate new situations is required. In their book on quantitative decisions in the pharmaceutical industry, ChuangStein and Kirby [11] generated most of the numerical results with simulations, even in cases where analytical results might have been possible to obtain. The modelling framework that we propose in this paper is explicitly developed to allow for utmost flexibility and an applicability to a very wide range of situations and questions. The framework also emphasizes the need to take a holistic view on the entire drug development program, which render attempts for analytical solutions problematic. It has consequently been an obvious choice to adapt the simulation approach when obtaining numerical results and analyses in this framework.

In this article we focussed on decisions made within a drug development project. For all medium and large size pharmaceutical companies, each drug project will constitute a part of a larger portfolio of projects. Thus, the planning and decision-making at the portfolio level is obviously of great importance [20, 27]. Although we have chosen here to focus on the project level, many of the underlying concepts would be applicable also to the portfolio. The choices to be made and the decisions to be supported may differ between the project and portfolio level, but the main pillars of ‘embracing uncertainty’ and ‘adapting a holistic approach’ remain valid in both cases.

## Supporting information

S1 File Model parameter values used in the illustrated example. (PDF)

## References

[1] Paul SM, Mytelka DS, Dunwiddie CT, Persinger CC, Munos BH, Lindborg SR, Schacht AL. How to improve R&D productivity: the pharmaceutical industry’s grand challenge. Nature Reviews Drug Discovery. 2010;9:203–214. 10.1038/nrd3078 20168317

[2] DiMasi JA, Grabowski HG, Hansen RW. Innovation in the pharmaceutical industry: New estimates of R&D costs. Journal of Health Economics. 2016;47: 20–33. 10.1016/j.jhealeco.2016.01.012 26928437

[3] FDA, U.S. Food and Drug Administration. Novel Drug Approvals for 2018. https://www.fda.gov/drugs/developmentapprovalprocess/druginnovation/ucm592464.htm. Source accessed: 20 Jan 2019.

[4] Mullard A. Industry R&D returns slip. Nature Reviews Drug Discovery. 2016;15:7. 10.1038/nrd.2016.135 27357013

[5] Deloitte. 2018. Unlocking R&D productivity: Measuring the return of pharmaceutical innovation 2018. https://www2.deloitte.com/us/en/pages/life-sciences-and-health-care/articles/measuring-return-from-pharmaceutical-innovation.html. Source accessed: 20 Jan 2019.

[6] Hay M, Thomas DW, Craighead JL, Economides C, Rosenthal J. Clinical development success rates for investigational drugs. Nature Biotechnology. 2014;32: 40–51. 10.1038/nbt.2786 24406927

[7] Mullard A. New drugs cost US$2.6 billion to develop. Nature Reviews Drug Discovery. 2014;13:877.

[8] David E, Tramontin T, Zemmel R. Pharmaceutical R&D: the road to positive returns. Nature Reviews Drug Discovery. 2009;8:609–610. 10.1038/nrd2948 19644471

[9] Fleming S. Pharma’s Innovation Crisis, Part 1: Why The Experts Can’t Fix It. https://www.forbes.com/sites/stanfleming/2018/09/06/why-experts-cant-fix-pharmas-innovation-crisis-part-1-and-what-to-do-about-it-part-2/#206b3db116fe. Source accessed: 20 Jan 2019.

[10] Cowlrick I, Hedner T, Wolf R, Olausson M, Klofsten M. Decision-making in the pharmaceutical industry: analysis of entrepreneurial risk and attitude using uncertain information. R&D Management. 2011;41: 321–336

[11] Chuang-Stein C, Kirby S. Quantitative Decisions in Drug Development., Springer Series in Pharmaceutical Statistics. 2017, ISBN 9783319460765.

[12] Antonijevic Z. Optimization of Pharmaceutical R&D Programs and Portfolios. Springer International Publishing Switzerland 2015, ISBN 978-3-319-09074-0

[13] Julious SA, Swank DJ. Moving statistics beyond the individual clinical trial: applying decision science to optimize a clinical development plan. Pharmaceutical Statistics. 2005;4: 37–46. 10.1002/pst.149

[14] Miller F, Burman CF. A decision theoretical modeling for Phase III investments and drug licensing. Journal of Biopharmaceutical Statistics. 2018;28: 698–721. 10.1080/10543406.2017.1377729 28920757

[15] Nixon R, Ireland B. Using decision analysis to support the design of clinical trials within a program In: *Optimization of Pharmaceutical R&D Programs and Portfolios*, Antonijevic Z, ed. Springer International Publishing Switzerland 2015, ISBN 978-3-319-09074-0.

[16] Boyer S, Brealey C, Davis AM. Attrition in Drug Discovery and Development In: Attrition in the Pharmaceutical Industry. 2015 John Wiley & Sons, Inc.

[17] Chen C, Beckman RA, Sun LZ. Maximizing return on investment in Phase II proof-of-concept trials In: *Optimization of Pharmaceutical R&D Programs and Portfolios*, Antonijevic Z, ed. Springer International Publishing Switzerland 2015, ISBN 978-3-319-09074-0.

[18] Antonijevic Z. Need for optimal design of pharmaceutical programs and portfolios in modern medical product development In: *Optimization of Pharmaceutical R&D Programs and Portfolios*, Antonijevic Z, ed. Springer International Publishing Switzerland 2015, ISBN 978-3-319-09074-0.

[19] Patel NR, Bolognese J, Chuang-Stein C, Hewitt D, Gammaitoni A, Pinheiro J. et al 2012. Designing Phase 2 Trials Based on Program-Level Considerations: A Case Study for Neuropathic Pain. Drug Information Journal. 2012; 46: 439–454

[20] Patel NR, Ankolekar S, Antonijevic Z, Rajicicd N. A mathematical model for maximizing the value of phase 3 drug development portfolios incorporating budget constraints and risk Statistics in medicine, 2013;32: 1763–1777. 10.1002/sim.5731 23300097

[21] Antonijevic Z, Pinheiro J, Fardipour P, Lewis RJ. Impact of Dose Selection Strategies Used in Phase II on the Probability of Success in Phase III. Statistics in Biopharmaceutical Research. 2010; 2: 469–486

[22] O’Hagan A, Stevens JW, Campbell MJ. Assurance in clinical trial design. Pharmaceutical Statistics. 2005;4: 187–201.

[23] Saint-Hilary G, Robert V, Gasparini M. Decision‐making in drug development using a composite definition of success. Pharmaceutical Statistics. 2018;17:555–569. 10.1002/pst.1870 29956453

[24] Marques CM, Moniz S, Pinho de Sousa J. Strategic decision-making in the pharmaceutical industry: A unified decision-making framework. Computers and Chemical Engineering. 2018;119: 171–189.

[25] Marques CM, Moniz S, Pinho de Sousa J, Barbosa-Póvoac AP. A simulation-optimization approach to integrate process design and planning decisions under technical and market uncertainties: A case from the chemical-pharmaceutical industry. Computers and Chemical Engineering. 2017;106:796–813.

[26] Moniz S, Barbosa-Póvoac AP, Pinho de Sousa J. On the complexity of production planning and scheduling in the pharmaceutical industry: the Delivery Trade-offs Matrix. In: Gernaey KV, Huusom JK, Gani R (Eds.), 12th International Symposium on Process Systems Engineering and 25th European Symposium on Computer Aided Process Engineering. 2015:1865–1870

[27] Jekunen A. Decision-making in product portfolios of pharmaceutical research and development–managing streams of innovation in highly regulated markets. Drug design, development and therapy. 2014;8:2009–2016. 10.2147/DDDT.S68579 25364229 PMC4211845

