# Supplementary material for: A modelling framework for improved design and decision-making in drug development
Source: PLoS One. 2019 Aug 28;14(8):e0220812. doi: 10.1371/journal.pone.0220812 (PMC6713335; doi:10.1371/journal.pone.0220812)
Supplement: S1 File — (PDF) [file pone.0220812.s001.pdf]

Table S1. Model parameter values used in the illustrated example; Time and cost of the five clinical trials included in the development program.

	Phase 1 trial	Expansion trial, part A	Expansion trial, part B	Accelerated pivotal trial	Standard development registration trial	Confirmatory trial
**Fixed cost, C_j^0**	1.5	1	1	3 - 5	5 - 10	5 - 10
**Cost per patient, C_j^N**	0.08 - 0.11	0.08 - 0.11	0.08 - 0.11	0.08 - 0.11	0.1 - 0.12	0.1 - 0.12
**Fixed time, T_j^0**	0.4	0.2	0.2	0.3	1 - 1.5	1 - 1.5
**Recruitment rate (patients per year), R_j**	40-60	40-60	60 - 80	150 - 200	250 - 350	250 - 350
**Number of patiens (per treatment arm), N**	20	20	20	100	300	300
**Number of treatment arms, A_j**	1	1	1	2	2	2

Note: Costs are given in MUSD. Times are given in years.
The parameters in the table are defined in Eqs (2) and (3) in Sec. 3.3. Some parameters are given as ranges to reflect relevant uncertainty.
Model parameters for registration activities are assumed to be Time=1 yr, Cost=2 MUSD (not included in the table).

Table S2. Model parameter values used in the illustrated example; Clinical effect model, CEM.

	Expansion trial, part A	Expansion trial, part B	Accelerated pivotal trial	Standard development registration trial	Confirmatory trial
**Endpoint**	Response rate	Response rate	Response rate ratio	Hazard ratio	Hazard ratio
**Distribution of true effect, E_j**	logN(-1.5 , 0.5)	logN(-1.5 , 0.5)	Active: logN(-1.5 , 0.5)<br>Control: logN(-1.3 , 0.1)	1- logN(-1.9 , 0.6)	1- logN(-1.9 , 0.6)
**Correlation, ρ_j**	0.8	0.8	0.8	0.9	0.9

Note: The distributions of E_j, and the parameter, ρ_j, are defined in Sec. 3.2.
No CEM is built for the first part of the Phase 1 trial, as efficacy is not evaluated in these patients

Table S3. Model parameter values used in the illustrated example; Decision criteria.

	After expansion, part A	After expansion, part B	After accelerated pivotal trial	After standard development trial	After registration process
**Decision criteria**	Omit part B and go directly to accelerated development if: #OR ≥ 8	Stop if: #OR ≤ 4<br><br>Go to standard development if: 5 ≤ #OR ≤ 15<br><br>Go to accelerated development if: #OR ≥ 16	Go to registration if ΔORR > 0,15<br><br>Stop otherwise	Go to registration if hazard ratio is statistically significant, i.e. $\hat{z}_j > 1.96$<br><br>Stop otherwise	Go to launch with probability 0.9

Note: The notation #OR is used to represent the observed number of overall responders in the expansion trials. ΔORR is the improvement in overall response rate compared to control arm in the accelerated pivotal trial. In each case this corresponds to $\hat{E}_j$ as defined in Eq (1) of Sec. 3.2. The values for the decision criteria correspond to E_j^{crit} and z_j^{crit} as defined in Sec. 3.4.

Table S4. Model parameter values used in the illustrated example; Market model.

	Parameter value
**Peak year sales,** *PYS*	47 MUSD
**Length of ramp-up time,** *U*	5 years
**Proportion of residual sales,** *f*	5%
**Anticipated effect in nominal sales estimate,** $\boldsymbol{E_0}$	HR: 0.8
**Year of patent expiry,** $\boldsymbol{T_E}$	2036

Note: The parameters in the table are defined in Eqs (5) and (6) in Sec. 3.7
